# Revealing gut microbiota biomarkers associated with melanoma immunotherapy response and key bacteria-fungi interaction relationships: evidence from metagenomics, machine learning, and SHAP methodology

**DOI:** 10.3389/fimmu.2025.1539653

**Published:** 2025-03-18

**Authors:** Yuhang Zhou, Wenjie Han, Yun Feng, Yue Wang, Xiaolin Liu, Tao Sun, Junnan Xu

**Affiliations:** ^1^ Department of Breast Medicine 1, Cancer Hospital of China Medical University, Liaoning Cancer Hospital, Shenyang, China; ^2^ Department of Pharmacology, Cancer Hospital of China Medical University, Liaoning Cancer Hospital, Shenyang, China; ^3^ Department of Bioinformatics, Kanghui Biotechnology Co., Ltd., Shenyang, China; ^4^ Department of Breast Medicine, Cancer Hospital of Dalian University of Technology, Liaoning Cancer Hospital, Shenyang, China

**Keywords:** gut microbiota, melanoma, immunotherapy, biomarker, metagenomics, machine learning, SHAP methodology

## Abstract

**Introduction:**

The gut microbiota is associated with the response to immunotherapy in cutaneous melanoma (CM). However, gut fungal biomarkers and bacterial-fungal interactions have yet to be determined.

**Methods:**

Metagenomic sequencing data of stool samples collected before immunotherapy from three independent groups of European ancestry CM patients were collected. After characterizing the relative abundances of bacteria and fungi, Linear Discriminant Analysis Effect Size (LEfSe) analysis, Random Forest (RF) model construction, and SHapley Additive exPlanations (SHAP) methodology were applied to identify biomarkers and key bacterial-fungal interactions associated with immunotherapy responders in CM.

**Results:**

Diversity analysis revealed significant differences in the bacterial and fungal composition between CM immunotherapy responders and non-responders. LEfSe analysis identified 45 bacterial and 4 fungal taxa as potential biomarkers. After constructing the RF model, the AUC of models built using bacterial and fungal data separately were 0.64 and 0.65, respectively. However, when bacterial and fungal data were combined, the AUC of the merged model increased to 0.71. In the merged model, the following taxa were identified as important biomarkers: *Romboutsia*, *Endomicrobium*, *Aggregatilinea*, *Candidatus Moduliflexus*, *Colwellia*, *Akkermansia*, *Mucispirillum*, and *Rutstroemia*, which were associated with responders, whereas *Zancudomyces* was associated with non-responders. Moreover, the positive correlation interaction between *Akkermansia* and *Rutstroemia* is considered a key bacterial-fungal interaction associated with CM immunotherapy response.

**Conclusion:**

Our results provide valuable insights for the enrichment of responders to immunotherapy in CM patients. Moreover, this study highlights the critical role of bacterial-fungal interactions in CM immunotherapy.

## 1 Introduction

Melanoma, a type of skin cancer, has garnered significant attention due to its grim prognosis, pronounced invasiveness, and limited survival potential. Cutaneous melanoma (CM), which constitutes about 5% of all skin cancers, is a particularly aggressive form (1). Despite its relatively low incidence, it is responsible for a staggering 55,500 fatalities each year (2). Therefore, the development of effective treatment strategies is a critical priority in enhancing the survival rates of CM patients. Fortunately, the emergence of immunotherapy has marked a significant advancement in improving the prognosis for CM patients (3). However, a proportion of patients are still unable to derive substantial benefit from this treatment (4). Therefore, it is highly meaningful to screening potential responders to immunotherapy and guide non-responders to promptly receive alternative effective treatments.

The gut microbiota has been shown to actively participate in the host’s local and systemic inflammation (5, 6). Therefore, the gut microbiota is considered a promising biomarker and potential therapeutic target across various fields (7–9). Early studies have indicated that the gut microbiota and its metabolites maintain immune system homeostasis in the host by modulating immune cell responses and functions (10, 11). This endows them with the ability to enhance or counteract immunotherapy responses by promoting local and systemic inflammation or inducing an immunosuppressive phenotype. Current gut microbiota studies primarily focus on bacterial taxa, likely due to the higher abundance of bacteria in the gut. A study analyzed fecal samples collected from CM patients undergoing immune checkpoint inhibitor (ICI) treatment and identified *Bifidobacterium longum*, *Enterococcus faecium* and *Collinsella aerofaciens* as potential bacterial taxa associated with enhanced efficacy of PD-L1 inhibitors (12). Notably, gut fungi are an underappreciated biomarker. This perspective arises from the fact that gut bacteria constitute 99.9% of the entire gut microbiome, leading to the neglect of the role of gut fungi (13). However, a recent study has identified gut fungi as potential biomarkers associated with immunotherapy (14). Additionally, the gut fungus *Schizosaccharomyces octosporus* is capable of fermenting starch into short-chain fatty acids (SCFAs) in the bodies of immunotherapy responders, functionally further highlighting the role of gut fungi in immunotherapy (15). A recent study suggests that both fungal and bacterial roles should be considered in cancer research (16).

To further investigate gut bacterial and fungal biomarkers associated with immunotherapy response in CM patients, we collected three publicly available metagenomic datasets and performed Linear Discriminant Analysis (LDA) Effect Size (LEfSe) analysis, Random forest (RF) machine learning, and SHapley Additive exPlanations (SHAP) methodology. It will provide valuable insights for the enrichment of responders to immunotherapy in CM patients. Moreover, this study highlights the critical role of bacterial-fungal interactions in CM immunotherapy.

## 2 Materials and methods

### 2.1 Acquisition of metagenomic datasets

We retrieved three publicly available metagenomic sequencing datasets of stool samples from CM patients prior to immunotherapy from the National Center for Biotechnology Information Sequence Read Archive (NCBI-SRA) using the following accession numbers: PRJEB43119 (17), PRJNA399742 (12) and PRJNA915098 (18). The PRJEB43119 dataset includes 165 samples, consisting of 22 complete responses, 42 partial responses, 30 stable diseases, and 71 progressive diseases. In subsequent analyses, we classified complete responses, partial responses, and stable diseases as responders, while progressive diseases were classified as non-responders. The PRJNA399742 dataset includes 14 responders, 23 non-responders, and 1 unknown sample. All samples in the PRJNA915098 dataset are derived from responders. The three cohorts were all of European descent, which helped minimize the potential impact of ethnic heterogeneity on the results. PRJEB43119 will serve as the discovery cohort and training set for machine learning, while PRJNA399742 and PRJNA915098 will be used as the replication cohort and external test sets for machine learning.

### 2.2 Data processing

To remove low-quality base sequences and adapters, the fastq software (v0.21.0) was used for quality control of the raw sequencing data. Subsequently, the clumpify software (v38.90) will be used for duplicate removal in the data. Based on the human reference genome (GRCh38.p13), Bowtie2 (v2.4.2) software was used to filter out host-derived reads to obtain clean sequence data. Next, MEGAHIT and QUAST were used to assemble the cleaned data and perform gene prediction. CD-HIT software was used to perform redundancy removal on the gene prediction results, resulting in a non-redundant initial gene catalogue. Clustering was performed using default parameters: identity set at 95% and coverage at 90%, with the longest sequence selected as the representative sequence. Finally, Salmon software was used to calculate the gene abundance information for each sample based on the Unigenes gene sequences and the clean data from each sample.

### 2.3 Diversity analysis

For alpha diversity, the indices assessed include Ace, Chao, Richness, and Shannon. The significance of inter-group differences between the two groups was assessed using the Wilcoxon rank-sum test. For beta diversity, principal components analysis (PCA) was performed based on the OTU table, followed by the creation of a scatter plot. The statistical significance was then assessed using analysis of similarity (ANOSIM). The analyses were performed using the “Vegan” and “ade4” packages in R software.

### 2.4 Differential analysis

Biomarker grouping was determined based on the effect sizes from LEfSe analysis (19). The differential analysis was performed using the Wilcoxon rank-sum test, and the results were adjusted for multiple hypothesis correction using the Bonferroni method (Bacterial comparisons: *P* < 0.0000327 is considered significant, and 0.0000327 < *P* < 0.05 is considered suggestive evidence. Fungal comparisons: *P* < 0.000568 is considered significant, and 0.000568 < *P* < 0.05 is considered suggestive evidence). The selection criteria were an LDA > 2 and a *P* < 0.05 in this exploratory study. The analysis was performed using the “LEfSe” package in R software.

### 2.5 Construction and test of the RF models

The diversity and complexity of gut microbiota species level data limit the reliability and robustness of the models. To simplify the data and enhance stability, we opted to construct the RF model at the genus level (20). The biomarkers identified by LEfSe analysis will be used as potential features for the RF model. The “rfcv” function in the “randomForest” package was used to compute the minimum error across different feature subsets using 10-fold cross-validation. Subsequently, to enhance the model’s reliability, we introduced an external test set and used the “pROC” package to plot the receiver operating characteristic (ROC) curve. To evaluate the model’s performance, we employed the area under the curve (AUC), calibration curve, and clinical decision curve analysis (DCA). The accuracy, sensitivity, specificity, and other performance metrics were calculated and reported for test sets.

### 2.6 SHAP methodology

SHAP values explain the contribution of each feature to the model’s prediction, whether it be positive or negative (21). The feature importance plot is used to display the features that have the greatest impact on the model’s predictions, with feature importance ranked based on the mean absolute SHAP values. The hive plot illustrates the direction of the impact of changes in feature relative abundance on the model’s predictions, which helps us understand the decision-making process within the complex model and further validate the results of LEfSe. This process utilizes the “shapviz” package to interpret the predictions of the RF model.

### 2.7 Bacterial-fungal interaction analysis

After identifying the key bacterial and fungal taxa, Spearman’s correlation coefficient was used to assess the key bacterial-fungal interactions in the three cohorts (22). The “stats” package was used to compute the correlations.

### 2.8 Statistics analysis

All statistical analysis and data visualization were conducted using R version 4.3.0.

## 3 Results

### 3.1 Changes in gut bacterial composition in CM responders

**Figure 1:**
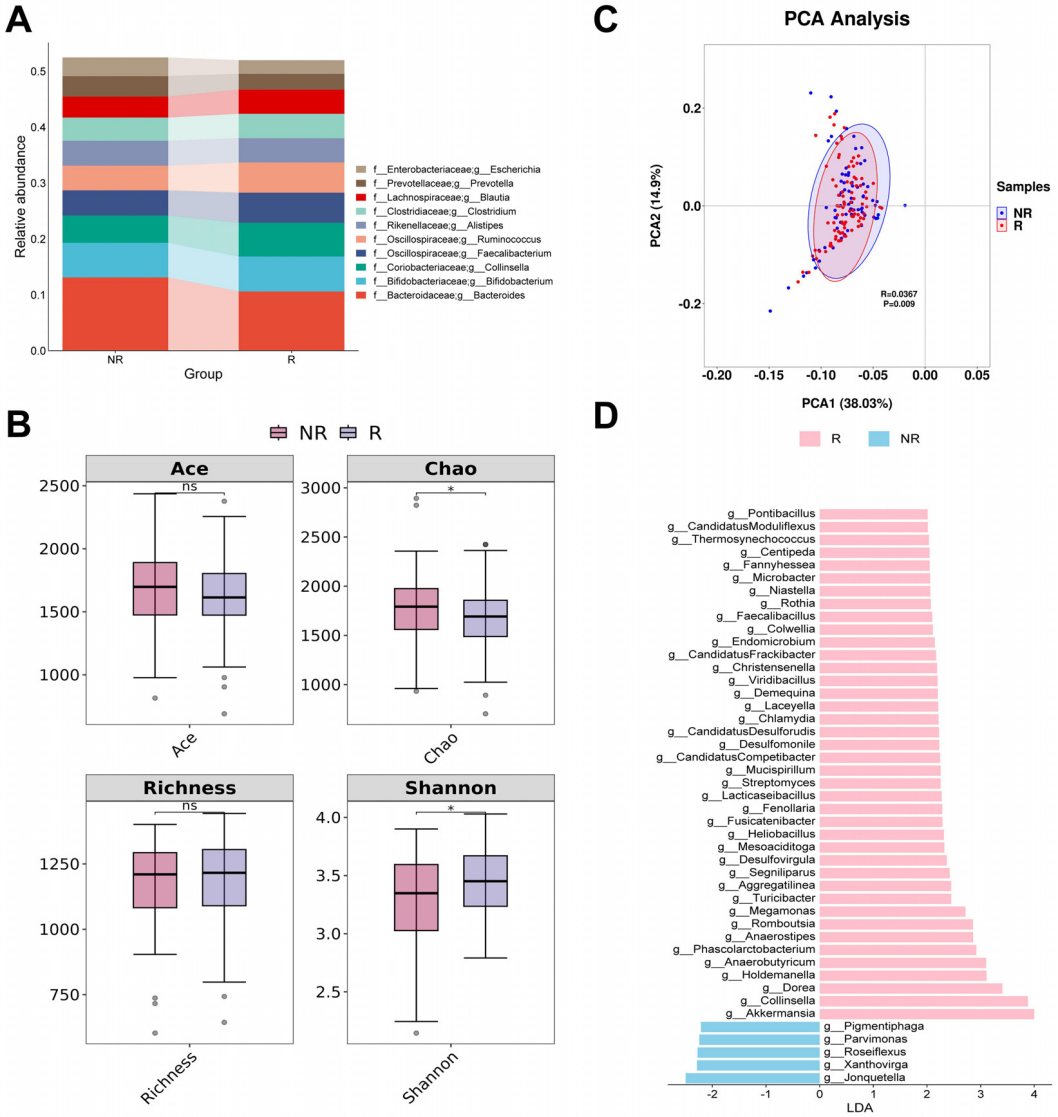
Analysis of differences in gut bacterial diversity and composition between responders and non-responders. **(A)** The top 10 taxa at the genus level based on relative abundance. **(B)** Alpha diversity analysis using Ace, Chao, Richness, and Shannon indices. **(C)** Beta diversity analysis using PCA. **(D)** Bar chart of the distribution of LDA values (LDA > 2). PCA, principal components analysis; LDA, linear discriminant analysis. “f_” and “g_” are family and genus respectively. *, P < 0.05; ns, not significant.

The four most abundant bacterial genera in responders and non-responders were Bacteroides, Bifidobacterium, Collinsella, and Fecalibacterium (**Figure 1A**). In the alpha diversity analysis, both the Ace and Richness indices did not show a significant statistical difference (P > 0.05). The Chao index of responders was significantly lower than that of non-responders, suggesting that the diversity of responders may be lower. However, the Shannon index of responders was significantly higher than that of non-responders, indicating a higher evenness in the gut bacterial composition of responders (**Figure 1B**). PCA analysis showed that the first two principal components explained approximately 52.93% of the diversity. Although the confidence intervals of the two groups partially overlap, ANOSIM analysis revealed a significant difference between responders and non-responders (R = 0.0367, P < 0.05) (**Figure 1C**). LEfSe analysis identified 45 potential biomarkers, including 40 bacterial taxa associated with responders and 5 bacterial taxa associated with non-responders. In responders, the top three bacterial taxa identified by LDA analysis were *Akkermansia*, *Collinsella*, and *Dorea*, while in non-responders, the top three were *Jonquetella*, *Xanthovirga*, and *Roseiflexus* (**Figure 1D**; **Supplementary Table S1**).

### 3.2 Bacterial feature selection and interpretation of the RF model

**Figure 2:**
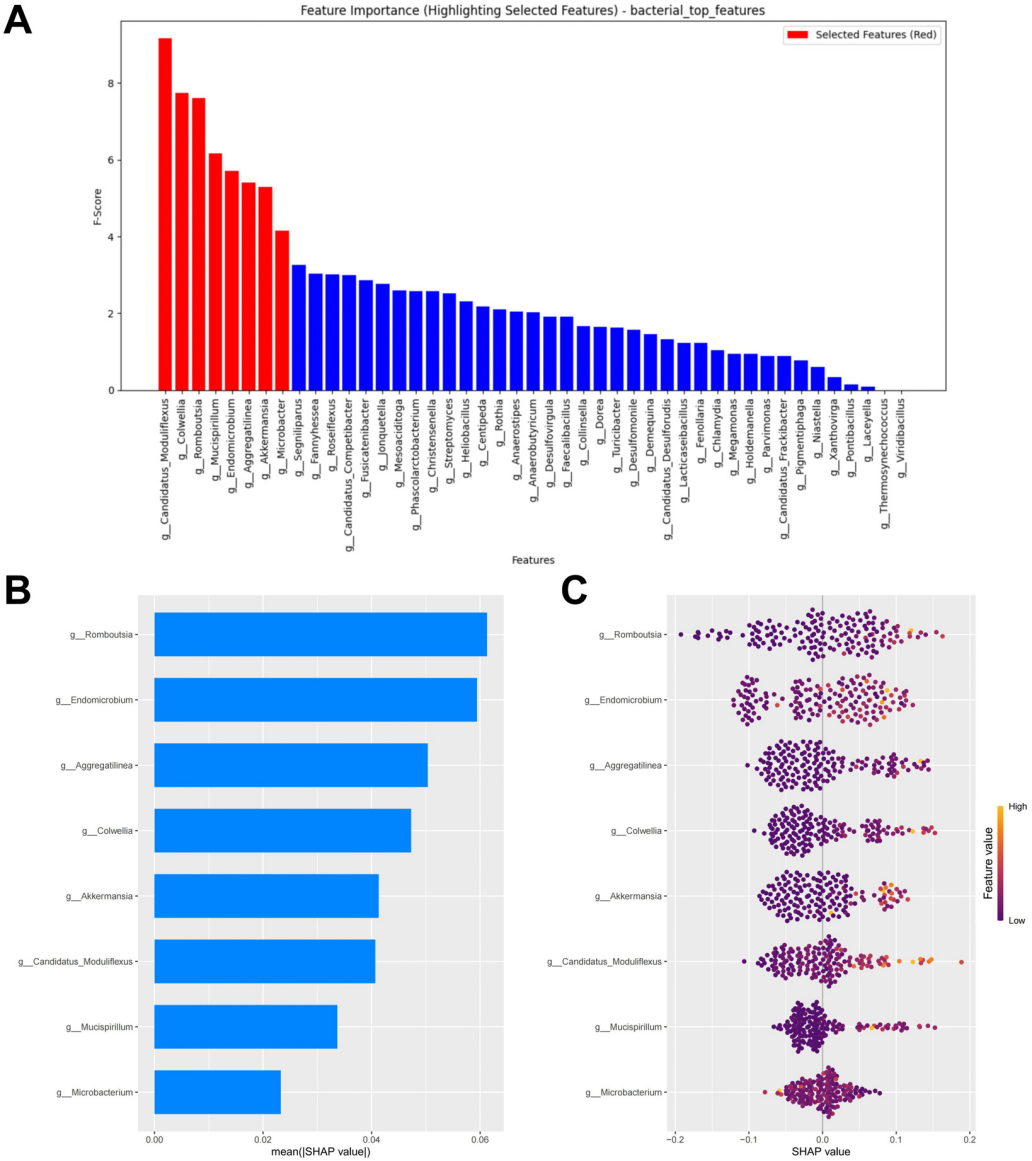
Bacterial feature selection and SHAP methodology. **(A)** Importance ranking computed by the RF algorithm. **(B)** Importance chart of SHAP variables. **(C)** Hive plot of SHAP variables. RF, random forest; SHAP, SHapley Additive exPlanations. “g_” is genus.

At the minimum error, the top eight biomarkers ranked by feature importance using the RF algorithm were included in the construction of the diagnostic model (**Figure 2A**). The SHAP summary plot of the model shows the effect of features on the prediction model (**Figure 2B**). The selected features were ranked from highest to lowest based on their average absolute SHAP values. From high to low are: *Romboutsia*, *Endomicrobium*, *Aggregatilinea*, *Colwellia*, *Akkermansia*, *Candidatus Moduliflexus*, *Mucispirillum*, and *Microbacter*. The hive plot shows that as the feature value (Relative abundance) of all features increases, their SHAP values tend to predict responders (**Figure 2C**). It is worth noting that these features were identified as responder associated bacterial taxa in the LEfSe analysis, further highlighting their potential value in predicting immunotherapy responses in CM patients.

### 3.3 Bacterial RF model performance evaluation

**Figure 3:**
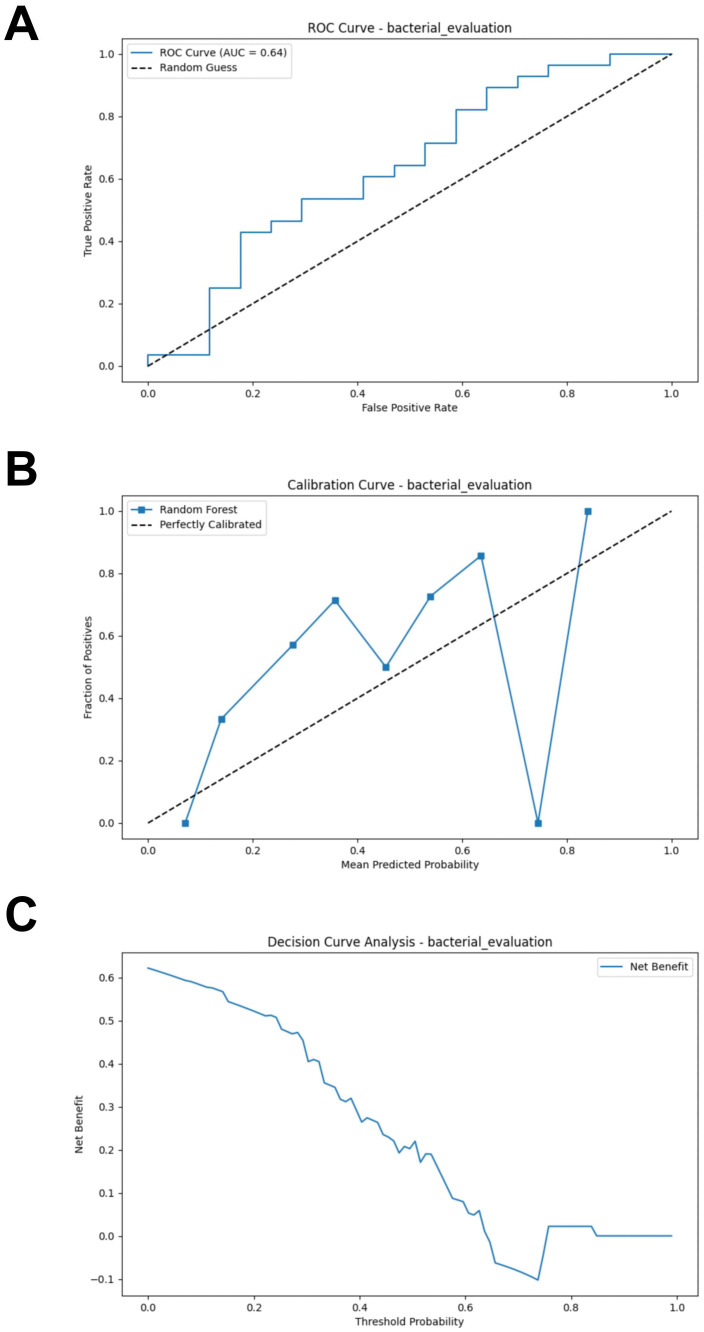
External validation of the bacterial model. **(A)** ROC curve of model in test set. **(B)** Calibration curve of the model on the test set. **(C)** Decision curve of the model on the test set. ROC, receiver operating characteristic.

The test set, composed of two cohorts, was used to evaluate the performance of the RF model. Using the test set, the RF model demonstrated poor performance (AUC = 0.637, Accuracy = 0.578, Sensitivity = 0.536, Specificity = 0.647, F1 Score = 0.584) (**Figure 3A**; **Supplementary Table S2**). We evaluated the accuracy of the RF model in predicting the response of CM patients to immunotherapy by analyzing the calibration curve and DCA. The calibration curve of the test set showed some degree of bias, reflecting the model’s generalization ability to unseen data (**Figure 3B**). The DCA analysis indicated that the model provided limited net benefit for clinical decision-making across most threshold probabilities (**Figure 3C**).

### 3.4 Changes in gut fungal composition in CM responders

**Figure 4:**
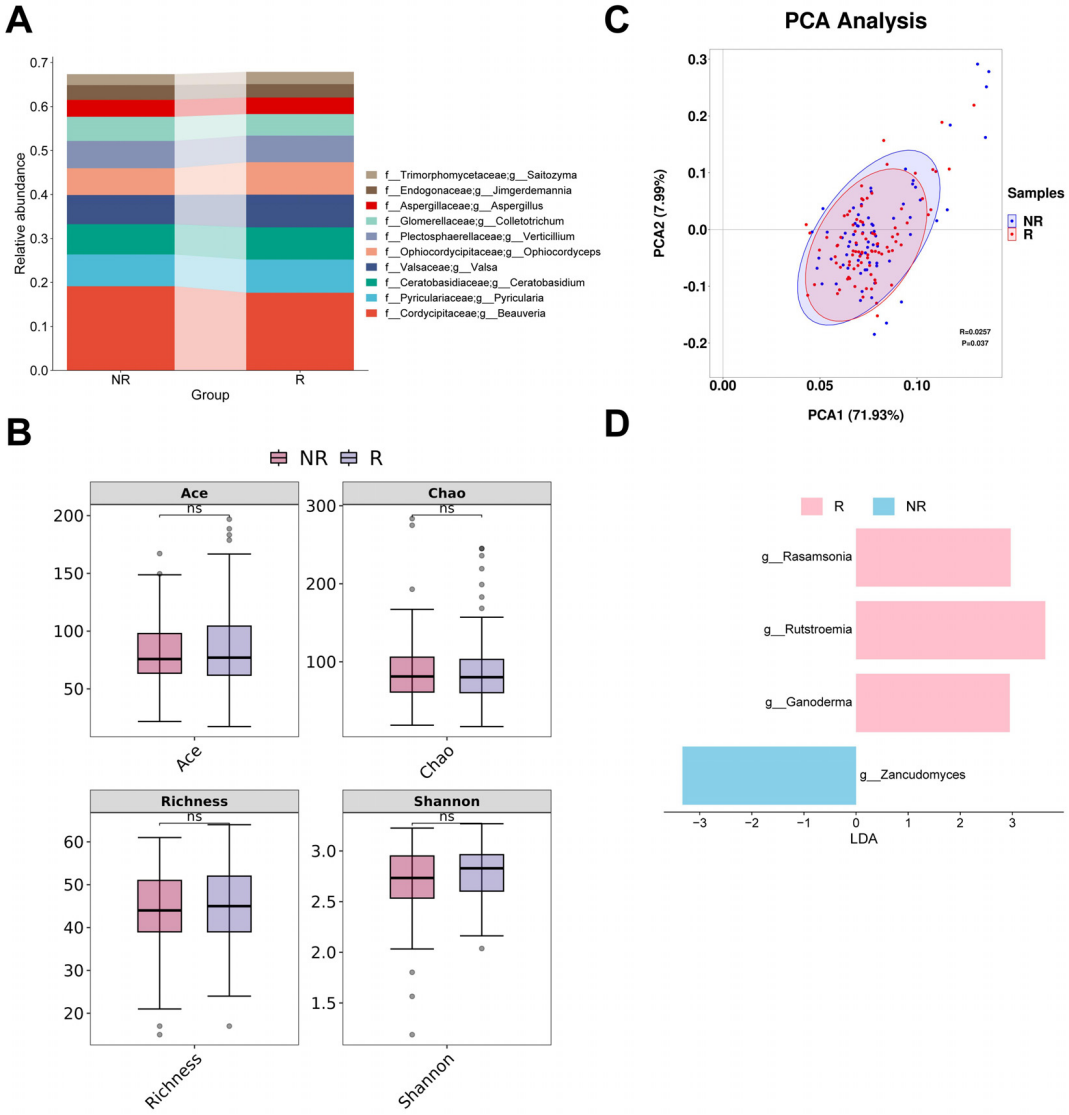
Analysis of differences in gut fungal diversity and composition between responders and non-responders. **(A)** The top 10 taxa at the genus level based on relative abundance. **(B)** Alpha diversity analysis using Ace, Chao, Richness, and Shannon indices. **(C)** Beta diversity analysis using PCA. **(D)** Bar chart of the distribution of LDA values (LDA > 2). PCA, principal components analysis; LDA, linear discriminant analysis. “f_” and “g_” are family and genus respectively. ns, not significant.

The four most abundant fungal genera in responders and non-responders were *Beauveria*, *Pyricularia*, *Ceratobasidium* and *Valsa* (**Figure 4A**). None of the four alpha diversity indices showed significant differences between groups (**Figure 4B**). PCA analysis revealed that the first two principal coordinates captured approximately 79.92% of the diversity. The confidence intervals of the two groups almost overlapped. However, the ANOSIM analysis revealed a significant difference between the groups (R = 0.0257, P < 0.05) (**Figure 4C**). The subsequent LEfSe analysis identified four potential gut fungal biomarkers, among which *Rasamsonia*, *Rutstroemia*, and *Ganoderma* were associated with responders, while *Zancudomyces* was exclusively associated with non-responders (**Figure 4D**; **Supplementary Table S3**).

### 3.5 Fungal feature selection and interpretation of the RF model

**Figure 5:**
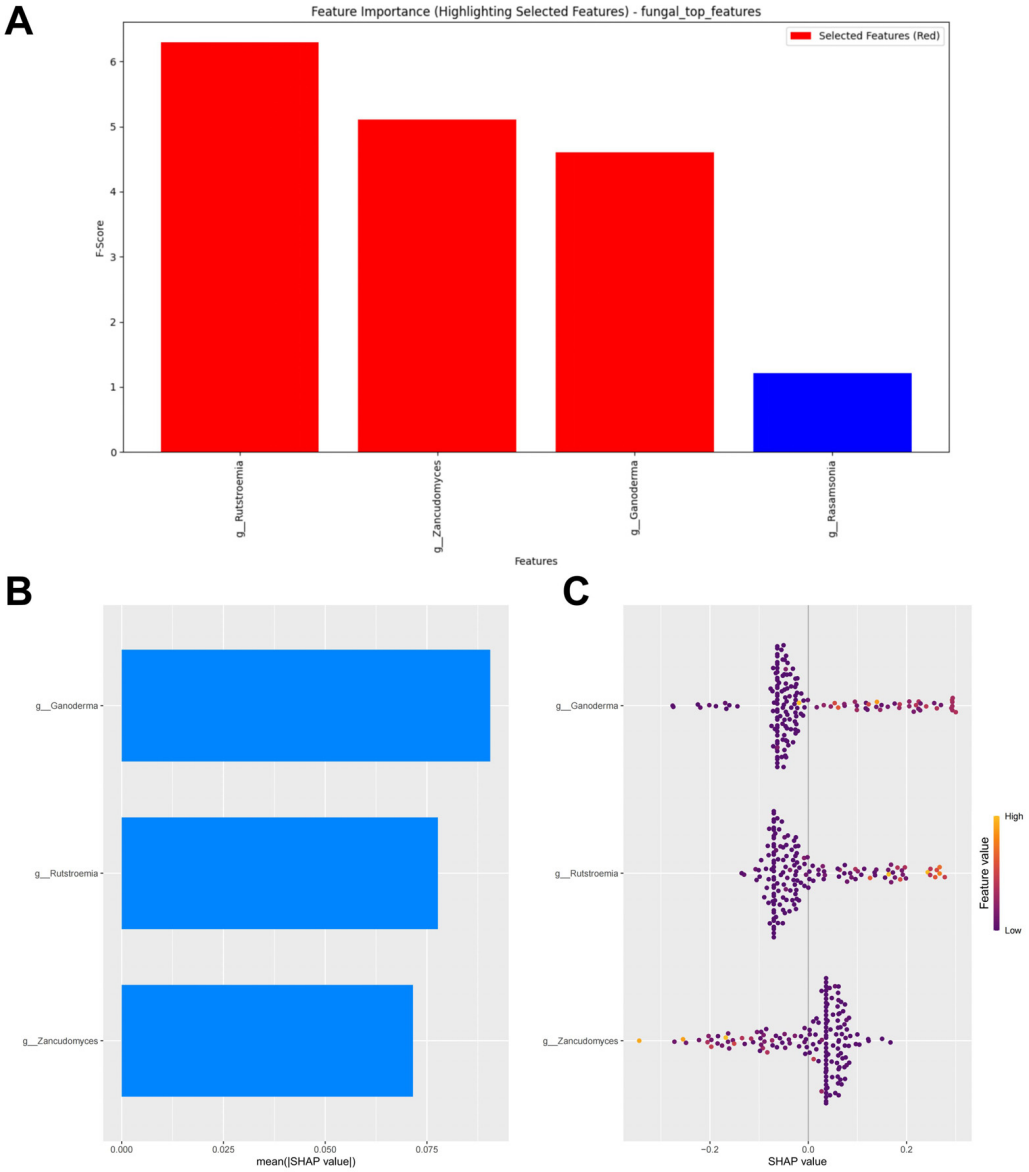
Fungal feature selection and SHAP methodology. **(A)** Importance ranking computed by the RF algorithm. **(B)** Importance chart of SHAP variables. **(C)** Hive plot of SHAP variables. RF, random forest; SHAP, SHapley Additive exPlanations. “g_” is genus.

When the error was minimized, the top three potential fungal biomarkers, ranked by importance based on the RF algorithm, were incorporated into the model construction (**Figure 5A**). The SHAP summary plot of the model illustrates the influence of the features on the prediction (**Figure 5B**). The selected features were ranked from highest to lowest based on their average absolute SHAP values. From high to low are: *Ganoderma, Rutstroemia* and *Zancudomyces*. The SHAP hive plot reveals that an increase in the feature value (Relative abundance) of *Ganoderma* and *Rutstroemia* is associated with predictions of responders, while an increase in the feature value of *Zancudomyces* is associated with predictions of non-responders, which is consistent with the results from the LEfSe analysis (**Figure 5C**).

### 3.6 Fungal RF model performance evaluation

**Figure 6:**
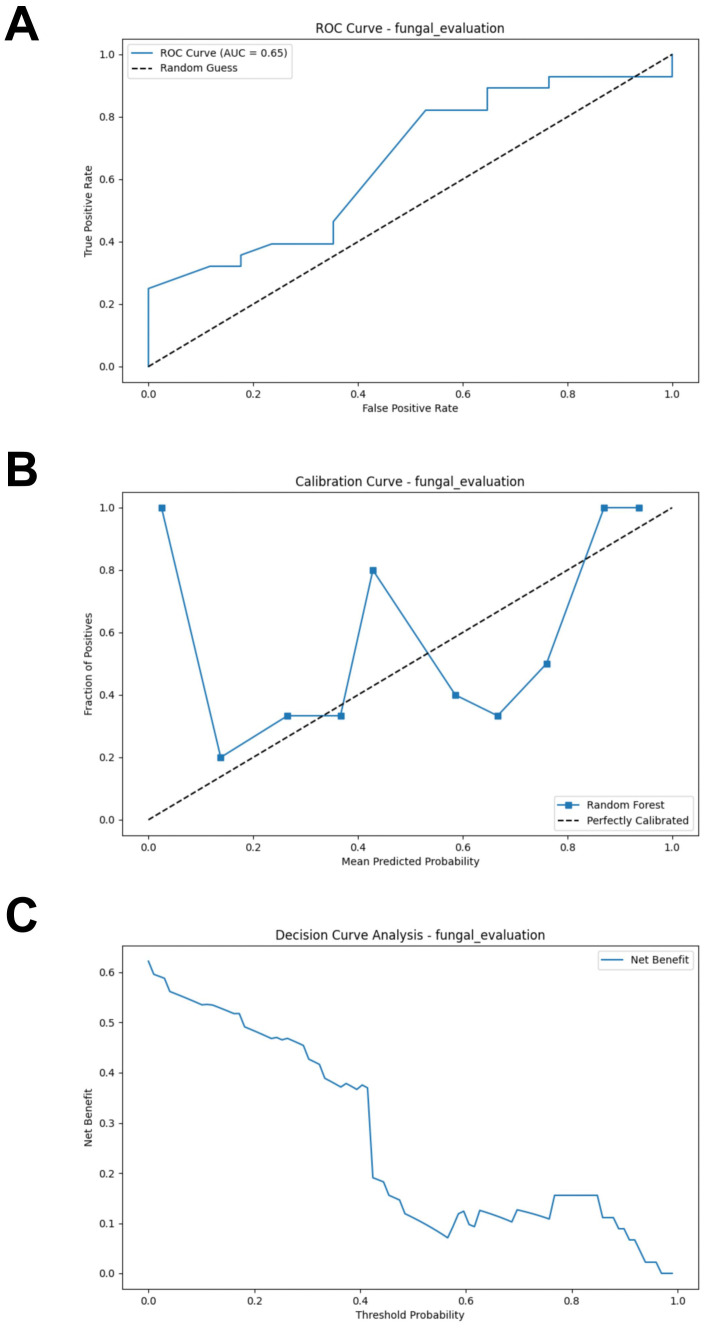
External validation of the fungal model. **(A)** ROC curve of model in test set. **(B)** Calibration curve of the model on the test set. **(C)** Decision curve of the model on the test set. ROC, receiver operating characteristic.

Similarly, a test set composed of data from two cohorts was used to evaluate the model’s predictive performance. Compared to the bacterial based model, the fungal RF model demonstrated better performance. However, its performance was generally suboptimal (AUC = 0.654, Accuracy = 0.489, Sensitivity = 0.393, Specificity = 0.647, F1 Score = 0.489) (**Figure 6A**; **Supplementary Table S4**). The calibration curve of the test set showed some degree of bias, reflecting the model’s generalization ability to unseen data (**Figure 6B**). The DCA analysis indicated that the model provided limited net benefit for clinical decision-making across most threshold probabilities (**Figure 6C**).

### 3.7 Merged feature selection and interpretation of the RF model

**Figure 7:**
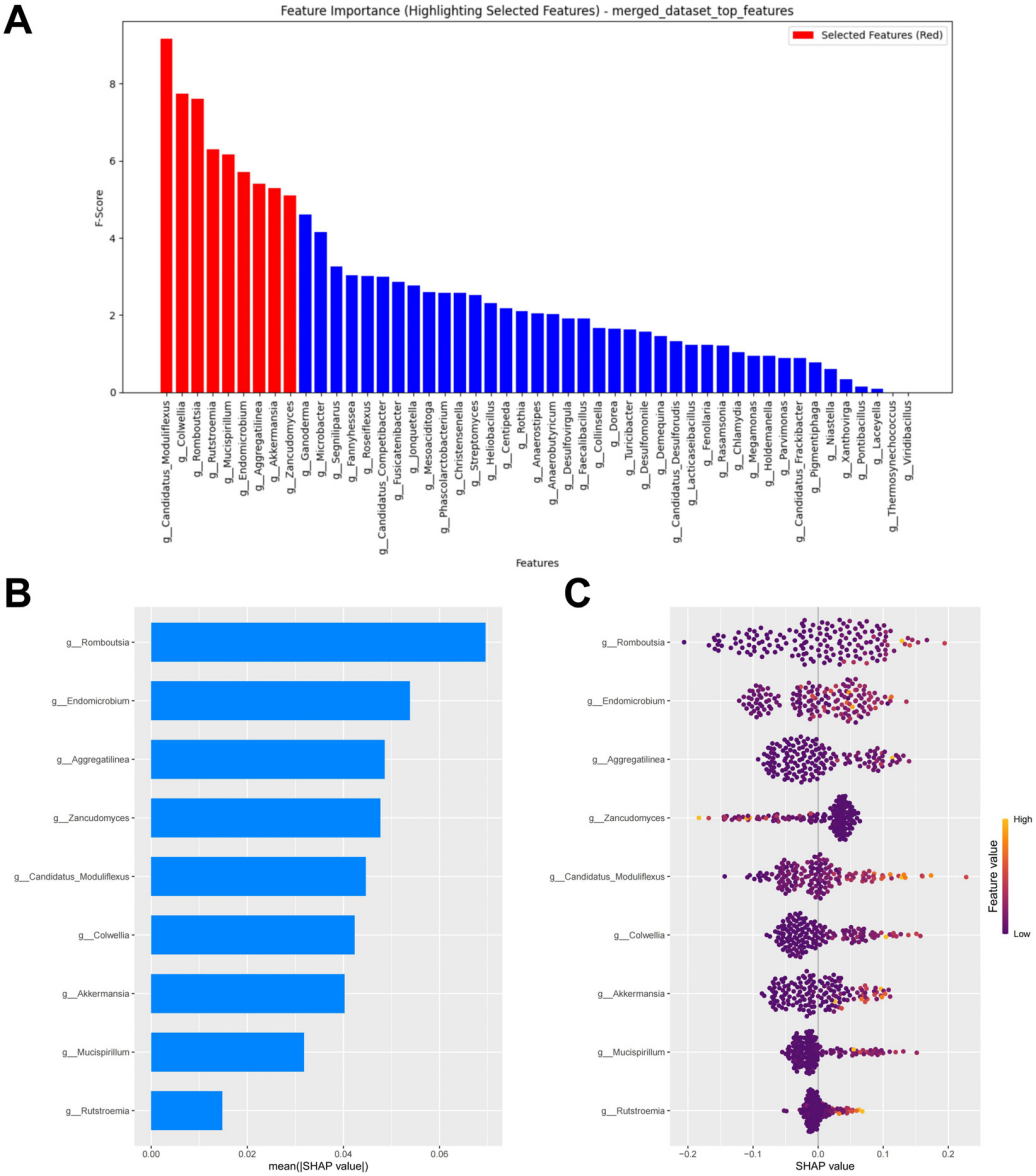
Merged feature selection and SHAP methodology. **(A)** Importance ranking computed by the RF algorithm. **(B)** Importance chart of SHAP variables. **(C)** Hive plot of SHAP variables. RF, random forest; SHAP, SHapley Additive exPlanations. “g_” is genus.

Models constructed separately based on bacteria or fungi both exhibited poor predictive performance. We further combined bacteria and fungi to explore the performance of the merged model. Under the condition of minimized error, the top nine potential biomarkers ranked by importance according to the RF algorithm were included in the model construction (**Figure 7A**). Among the biomarkers included in the model, seven bacterial biomarkers were identified, including *Candidatus Moduliflexus*, *Colwellia*, *Romboutsia*, *Mucispirillum*, *Endomicrobium*, *Aggregatilinea*, and *Akkermansia*. Only two fungal biomarkers were included in the model, namely *Rutstroemia* and *Zancudomyces*. The SHAP summary plot of the model illustrates the influence of the features on the prediction (**Figure 7B**). The selected features were ranked from highest to lowest based on their average absolute SHAP values. From high to low are: *Romboutsia*, *Endomicrobium*, *Aggregatilinea*, *Zancudomyces*, *Candidatus Moduliflexus*, *Colwellia*, *Akkermansia*, *Mucispirillum* and *Rutstroemia*. The SHAP hive plot reveals that an increase in the feature value (Relative abundance) of Zancudomyces is associated with the prediction of non-responders, while an increase in other feature values is associated with the prediction of responders, which is consistent with the results from the LEfSe analysis (**Figure 7C**).

### 3.8 Merged RF model performance evaluation

**Figure 8:**
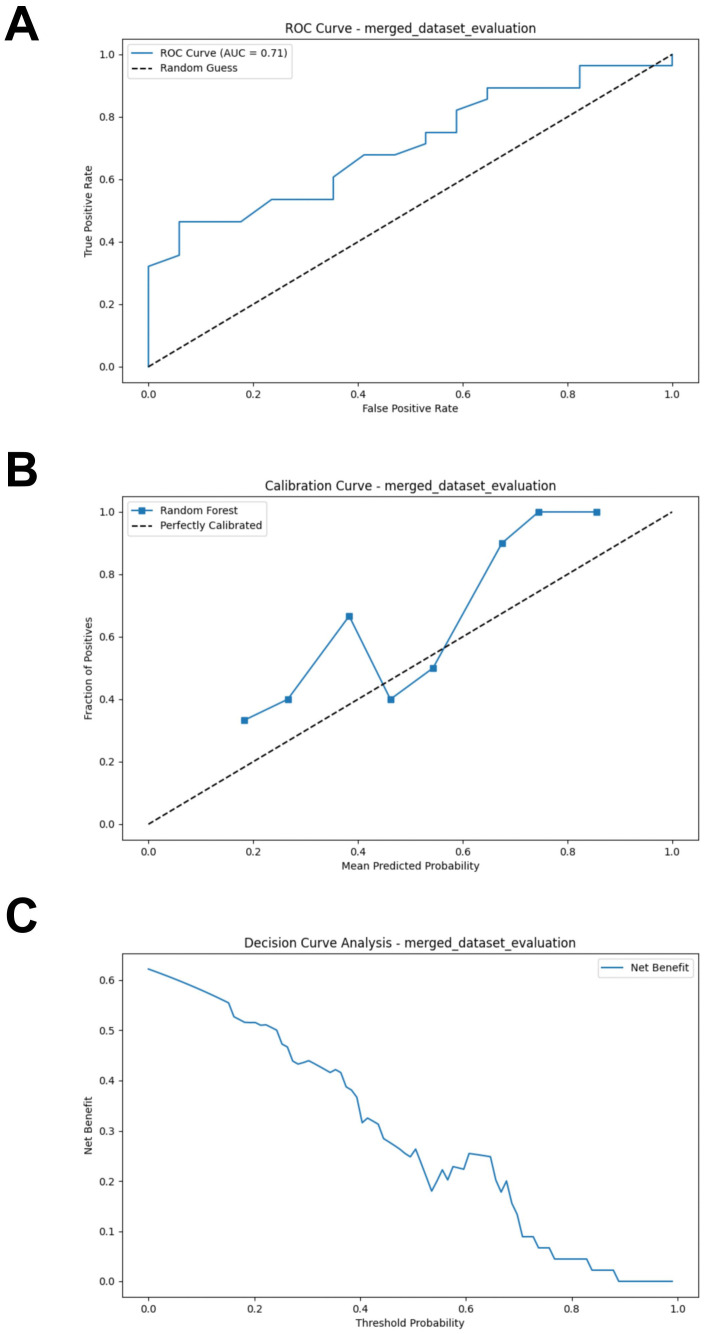
External validation of the merged model. **(A)** ROC curve of model in test set. **(B)** Calibration curve of the model on the test set. **(C)** Decision curve of the model on the test set. ROC, receiver operating characteristic.

After combining the bacterial and fungal data, the performance of the RF model showed a significant improvement compared to the models constructed separately using bacteria or fungi, with the following metrics: AUC = 0.707, Accuracy = 0.644, Sensitivity = 0.679, Specificity = 0.588, and F1 Score = 0.648 (**Figure 8A**; **Supplementary Table S5**). Compared to the previous models, the calibration curve of the merged model shows a better fit, indicating a higher consistency between the model predictions and the actual incidence (**Figure 8B**). DCA indicates that the merged model provides higher net benefit for clinical decisions across most threshold probabilities (**Figure 8C**). Thus, merging the data from gut bacteria and fungi provides a more comprehensive understanding of the gut microbiome and further supports the potential interactions between bacteria and fungi.

### 3.9 Bacterial-fungal interaction analysis

**Figure 9:**
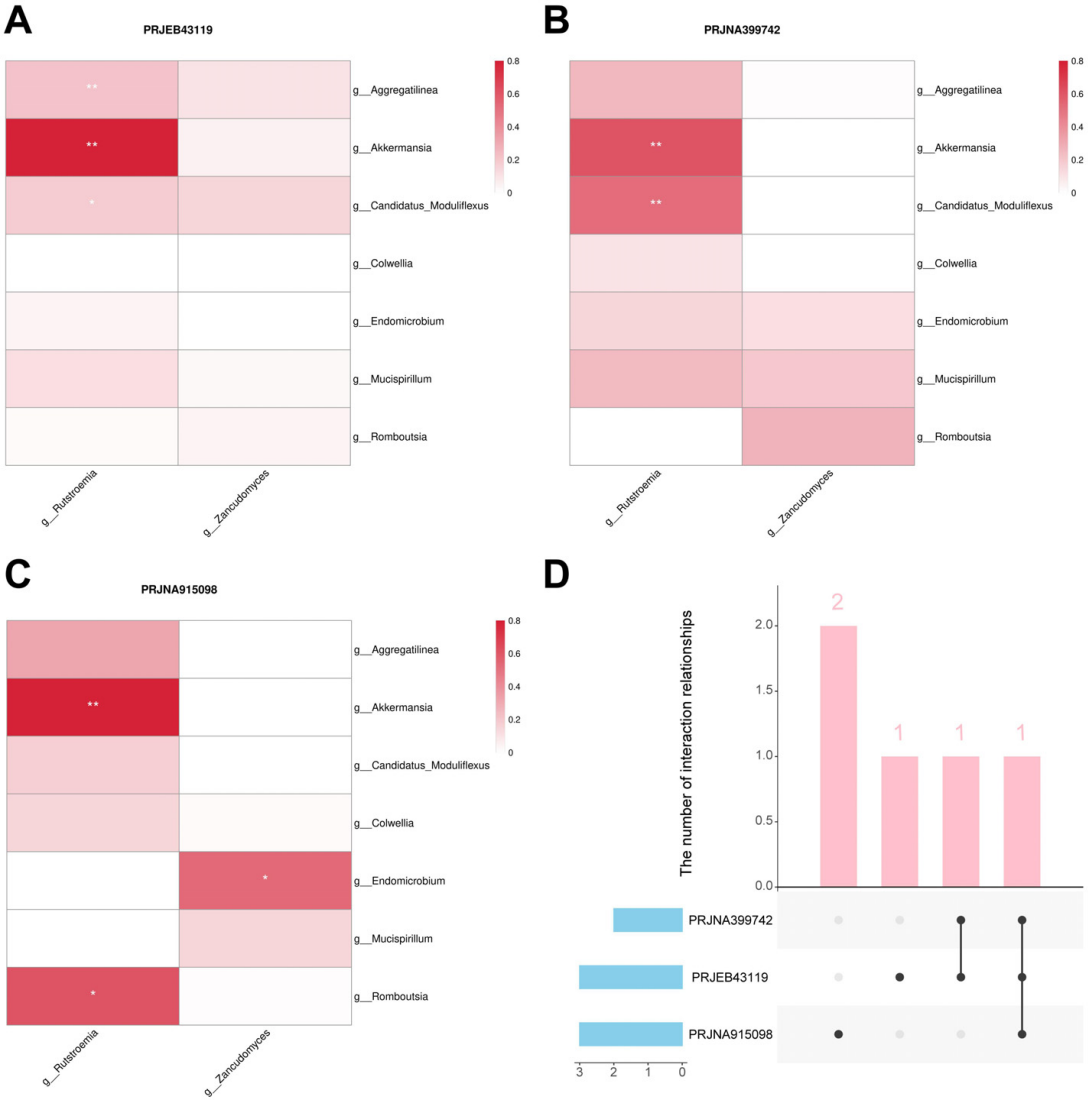
Bacteria-fungal interactions analysis. **(A)** Interactions in the PRJEB43119 dataset. **(B)** Interactions in the PRJNA399742 dataset. **(C)** Interactions in the PRJNA915098 dataset. **(D)** Upset plot of the three cohorts. “g_” is genus. *, P < 0.05; **, P < 0.01.

Bacteria and fungi involved in the construction of the merged model were identified as important biomarkers. To investigate the interactions between bacteria and fungi, we constructed a microbiome association network by calculating the Spearman correlation coefficient to identified key interactions. In the PRJEB43119, PRJNA399742, and PRJNA915098 datasets, three, two, and three positive correlations were identified, respectively (**Figures 9A–C**). The positive correlation interaction between *Akkermansia* and *Rutstroemia* was identified in all three cohorts, which may represent a key interaction associated with immunotherapy response in CM patients. Moreover, the positive correlation interaction between *Candidatus Moduliflexus* and *Rutstroemia* was identified in two cohorts, providing relatively strong evidence for it being a key interaction (**Figure 9D**).

## 4 Discussion

In previous studies, methods combining LEfSe and machine learning have been widely used to identify biomarkers (23–25). In this study, we employed the SHAP methodology to further validate the results of the LEfSe analysis, and provided a comprehensive evaluation of feature importance for bacterial-fungal interactions. Thus, we identified 9 important biomarkers associated with immunotherapy response in CM patients. Among these biomarkers, seven are gut bacteria, including *Romboutsia*, *Endomicrobium*, *Aggregatilinea*, *Candidatus Moduliflexus*, *Colwellia*, *Akkermansia*, and *Mucispirillum*, all of which are associated with responders. Two gut fungal biomarkers, *Rutstroemia* and *Zancudomyces*, are associated with responders and non-responders, respectively. Moreover, bacterial-fungal interaction analysis revealed a significant positive correlation between *Akkermansia* and *Rutstroemia* across all cohorts, which may represent a key bacterial-fungal interaction in immunotherapy for CM patients.

Early study has found that the gut bacterial alpha diversity of responders is significantly higher than that of non-responders, which is consistent with our current results (26). In addition, a significant difference in the beta diversity of gut fungi was observed between the durable clinical benefit group and the non-durable clinical benefit group in biliary tract cancer immunotherapy. However, no significant difference was found in the alpha diversity, as measured by the Shannon index (14). This is also consistent with our results. Currently, gut microbiota dysbiosis is widely recognized as a significant factor contributing to melanoma development (27, 28). Maintaining the balance of the gut microbiota plays a crucial role in the prevention, management, and treatment of melanoma. Among the bacterial biomarkers we have identified, *Akkermansia* has been extensively studied (29). A study has shown that *Akkermansia* plays a crucial role in promoting epithelial development mediated by intestinal stem cells and contributes to the maintenance of intestinal homeostasis (30). Epithelial development contributes to the establishment of an immune environment that supports the colonization of probiotics (31). Moreover, intestinal homeostasis regulates the gut microbiota and promotes its tendency towards balanced state (32). These findings suggest that *Akkermansia* may have complex interactions with other members of the gut microbiota. Meanwhile, a study reported that dihydroartemisinin can enhance the sensitivity of patients with hepatocellular carcinoma to immunotherapy by increasing the abundance of *Akkermansia* (33). This further underscores the potential of *Akkermansia* as a core biomarker associated with immunotherapy response. Our results provide strong evidence supporting the interaction between *Akkermansia* and *Rutstroemia*. Nevertheless, research on the relationship between Rutstroemia and human health remains limited.

Metabolites of the gut microbiota actively participate in the communication between the gut-skin axis. Among the biomarkers associated with responders that we identified, *Akkermansia* and *Mucispirillum* generate SCFAs by degrading gut mucin and fermenting carbohydrates, respectively. Immunotherapy exerts its antitumor effects primarily through the reactivation of immune cells (34). Propionate and butyrate, two types of SCFAs, have been widely recognized as contributing to the enhancement of the antitumor effects of immunotherapy. Both propionate and butyrate are capable of inhibiting histone deacetylases (HDACs) (35, 36). When HDAC is inhibited, histone acetylation of the PD-L1 gene increases, thereby enhancing the expression of the gene (37). In addition, a recent study indicated that butyrate increases histone acetylation levels in CD8+ T cells, thereby promoting the expression of PD-1/CD28 (38). HDAC inhibition can also prevent the infiltration of myeloid-derived suppressor cells (MDSCs) into tumors and reprogram the tumor’s immunosuppressive microenvironment (39). These mechanisms will contribute to enhancing the efficacy of immunotherapy in melanoma.

It should also be noted that a clinical study found that high levels of butyrate in the blood can inhibit the aggregation of memory T cells and ICOS+ CD4+ T cells, as well as IL-2 impregnation, induced by ipilimumab (40). The negative effects may be associated with the accumulation of Tregs. Studies have reported that both propionate and butyrate can promote the differentiation of Foxp3+ Tregs (41, 42). Interestingly, propionate and butyrate promote the differentiation of Foxp3+ Tregs through the inhibition of HDAC and upregulation of the Foxp3 enhancer (43). The enrichment of Tregs not only directly inhibits effector T cells but also suppresses antigen-presenting cells, thereby indirectly inhibiting the activation of effector T cells. This has devastating implications for immunotherapy. Therefore, when using butyrate-producing bacteria as biomarkers for immunotherapy response, it is necessary to further monitor the dynamic changes of Tregs.

Currently, biomarkers for predicting the efficacy of immunotherapy in CM patients are relatively lacking, whereas the composition and function of the gut microbiota play a crucial role in modulating immune responses and treatment outcomes, making it a potential biomarker for predicting immunotherapy efficacy and helping optimize therapeutic decision-making. It is worth noting that our study provides foundational insights into the role of the gut microbiota in CM immunotherapy. However, further research is needed to establish a direct causal relationship and determine whether our findings can guide interventions such as probiotics or fecal microbiota transplants (FMT).

This study presents several unique advantages. First, the use of metagenomic sequencing providing more precise taxonomic data compared to 16S rRNA. In addition, metagenomic sequencing also enables us to further explore the relationship between gut fungi and immunotherapy response in CM patients. However, certain limitations still need to be acknowledged. Our study focused on the genus level, lacking investigation at other taxonomic levels, which may result in the omission of some key information. In the future, our research will be expanded to include other taxonomic levels to complement the gaps in the current study. Moreover, our study population is focused on Europeans, which, although eliminating the impact of racial heterogeneity on the results, also limits the generalizability of our findings to other populations. In the future, efforts should be made to include a broader range of populations to ensure the generalizability of the results. Finally, due to the widespread lack of understanding of *Rutstroemia*’s functions, this study did not conduct an in-depth investigation into the biological functions of key interactions, which has also driven us to further explore the biological significance of the key interaction in the future.

Overall, due to the differences in gut microbiota composition and diversity between responders and non-responders, the gut microbiota can be utilized as a biomarker for immunotherapy response. These biomarkers will aid in enriching the population of immunotherapy responders among CM patients. Furthermore, the inclusion of the fungal component highlights the potential role of bacteria-fungi interactions in immunotherapy response.

## Data Availability

The original contributions presented in the study are included in the article/**Supplementary Material**. Further inquiries can be directed to the corresponding author/s.

## References

[1] Siegel RL Giaquinto AN Jemal A. Cancer statistics, 2024. CA Cancer J Clin. (2024) 74:12–49. doi: 10.3322/caac.21820 38230766

[2] SChadendorf D van Akkooi ACJ Berking C Griewank KG Gutzmer R Hauschild A. Melanoma. Lancet. (2018) 392:971–84. doi: 10.1016/S0140-6736(18)31559-9 30238891

[3] Huang AC Zappasodi R. A decade of checkpoint blockade immunotherapy in melanoma: understanding the molecular basis for immune sensitivity and resistance. Nat Immunol. (2022) 23:660–70. doi: 10.1038/s41590-022-01141-1 PMC9106900 35241833

[4] SChadendorf D Long GV Stroiakovski D Karaszewska B Hauschild A Levchenko E. Three-year pooled analysis of factors associated with clinical outcomes across dabrafenib and trametinib combination therapy phase 3 randomized trials. Eur J Cancer. (2017) 82:45–55. doi: 10.1016/j.ejca.2017.05.033 28648698

[5] Morrison DJ Preston T. Formation of short chain fatty acids by the gut microbiota and their impact on human metabolism. Gut Microbes. (2016) 7:189–200. doi: 10.1080/19490976.2015.1134082 26963409 PMC4939913

[6] Clemente JC Manasson J Scher JU. The role of the gut microbiome in systemic inflammatory disease. BMJ. (2018) 360:j5145. doi: 10.1136/bmj.j5145 29311119 PMC6889978

[7] Su Q Wong OWH Lu W Wan Y Zhang L Xu W. Multikingdom and functional gut microbiota markers for autism spectrum disorder. Nat Microbiol. (2024) 9:2344–55. doi: 10.1038/s41564-024-01739-1 38977906

[8] Heumel S de Rezende Rodovalho V Urien C Specque F Brito Rodrigues P Robil C. Shotgun metagenomics and systemic targeted metabolomics highlight indole-3-propionic acid as a protective gut microbial metabolite against influenza infection. Gut Microbes. (2024) 16:2325067. doi: 10.1080/19490976.2024.2325067 38445660 PMC10936607

[9] Glitza IC Seo YD Spencer CN Wortman JR Burton EM Alayli FA. Randomized placebo-controlled, biomarker-stratified phase Ib microbiome modulation in melanoma: impact of antibiotic preconditioning on microbiome and immunity. Cancer Discovery. (2024) 14:1161–75. doi: 10.1158/2159-8290.CD-24-0066 PMC11215408 38588588

[10] Thomas S Izard J Walsh E Batich K Chongsathidkiet P Clarke G. The host microbiome regulates and maintains human health: A primer and perspective for non-microbiologists. Cancer Res. (2017) 77:1783–812. doi: 10.1158/0008-5472.CAN-16-2929 PMC5392374 28292977

[11] Zhou Y Han W Feng Y Wang Y Sun T Xu J. Microbial metabolites affect tumor progression, immunity and therapy prediction by reshaping the tumor microenvironment (Review). Int J Oncol. (2024) 65:73. doi: 10.3892/ijo.2024.5661 38847233 PMC11173369

[12] Matson V Fessler J Bao R Chongsuwat T Zha Y Alegre M-L. The commensal microbiome is associated with anti-PD-1 efficacy in metastatic melanoma patients. Science. (2018) 359:104–8. doi: 10.1126/science.aao3290 PMC6707353 29302014

[13] Li L Huang X Chen H. Unveiling the hidden players: exploring the role of gut mycobiome in cancer development and treatment dynamics. Gut Microbes. (2024) 16:2328868. doi: 10.1080/19490976.2024.2328868 38485702 PMC10950292

[14] Zhu C Wang Y Zhu R Wang S Xue J Zhang D. Gut microbiota and metabolites signatures of clinical response in anti-PD-1/PD-L1 based immunotherapy of biliary tract cancer. biomark Res. (2024) 12:56. doi: 10.1186/s40364-024-00607-8 38831368 PMC11149318

[15] Huang X Hu M Sun T Li J Zhou Y Yan Y. Multi-kingdom gut microbiota analyses define bacterial-fungal interplay and microbial markers of pan-cancer immunotherapy across cohorts. Cell Host Microbe. (2023) 31:1930–43.e4. doi: 10.1016/j.chom.2023.10.005 37944495

[16] Wang Y Wang Y Zhou Y Feng Y Sun T Xu J. Tumor-related fungi and crosstalk with gut fungi in the tumor microenvironment. Cancer Biol Med. (2024) 21:977–94. doi: 10.20892/j.issn.2095-3941.2024.0240 39601429 PMC11667784

[17] Lee KA Thomas AM Bolte LA Björk JR de Ruijter LK Armanini F. Cross-cohort gut microbiome associations with immune checkpoint inhibitor response in advanced melanoma. Nat Med. (2022) 28:535–44. doi: 10.1038/s41591-022-01695-5 PMC8938272 35228751

[18] Golčić M Simetić L Herceg D Blažičević K Kenđel Jovanović G Dražić I. Analysis of the gut microbiome and dietary habits in metastatic melanoma patients with a complete and sustained response to immunotherapy. Cancers (Basel). (2023) 15:3052. doi: 10.3390/cancers15113052 37297014 PMC10252899

[19] Chang F He S Dang C. Assisted selection of biomarkers by linear discriminant analysis effect size (LEfSe) in microbiome data. J Vis Exp. (2022) 16:183. doi: 10.3791/61715 35635468

[20] Jiang L Cun Y Wang Q Wu K Hu M Wu Z. Predicting acute lung injury in infants with congenital heart disease after cardiopulmonary bypass by gut microbiota. Front Immunol. (2024) 15:1362040. doi: 10.3389/fimmu.2024.1362040 39512354 PMC11540645

[21] Fachet M, Mushunuri RV Bergmann CB Marzi I Hoeschen C Relja B. Utilizing predictive machine-learning modelling unveils feature-based risk assessment system for hyperinflammatory patterns and infectious outcomes in polytrauma. Front Immunol. (2023) 14:1281674. doi: 10.3389/fimmu.2023.1281674 38193076 PMC10773821

[22] Zou Y Wu Z Deng L Wu A Wu F Li K. cooccurNet: an R package for co-occurrence network construction and analysis. Bioinformatics. (2017) 33:1881–2. doi: 10.1093/bioinformatics/btx062 28174895

[23] Wang N Yang J Han W Han M Liu X Jiang L. Identifying distinctive tissue and fecal microbial signatures and the tumor-promoting effects of deoxycholic acid on breast cancer. Front Cell Infect Microbiol. (2022) 12:1029905. doi: 10.3389/fcimb.2022.1029905 36583106 PMC9793878

[24] Wang Y Wang Y Han W Han M Liu X Dai J. Intratumoral and fecal microbiota reveals microbial markers associated with gastric carcinogenesis. Front Cell Infect Microbiol. (2024) 14:1397466. doi: 10.3389/fcimb.2024.1397466 39355268 PMC11442432

[25] Han W Wang N Han M Liu X Sun T Xu J. Identification of microbial markers associated with lung cancer based on multi-cohort 16 s rRNA analyses: A systematic review and meta-analysis. Cancer Med. (2023) 12:19301–19. doi: 10.1002/cam4.6503 PMC10557844 37676050

[26] Gopalakrishnan V Spencer CN Nezi L Reuben A Andrews MC Karpinets TV. Gut microbiome modulates response to anti–PD-1 immunotherapy in melanoma patients. Science. (2018) 359:97–103. doi: 10.1126/science.aan4236 29097493 PMC5827966

[27] Woo YR Cho SH Lee JD Kim HS. The human microbiota and skin cancer. Int J Mol Sci. (2022) 23:1813. doi: 10.3390/ijms23031813 35163734 PMC8837078

[28] Mekadim C Skalnikova HK Cizkova J Cizkova V Palanova A Horak V. Dysbiosis of skin microbiome and gut microbiome in melanoma progression. BMC Microbiol. (2022) 22:63. doi: 10.1186/s12866-022-02458-5 35216552 PMC8881828

[29] Cani PD Depommier C Derrien M Everard A de Vos WM. Akkermansia muciniphila: paradigm for next-generation beneficial microorganisms. Nat Rev Gastroenterol Hepatol. (2022) 19:625–37. doi: 10.1038/s41575-022-00631-9 35641786

[30] Kim S Shin Y-C Kim T-Y Kim Y Lee Y-S Lee S-H. Mucin degrader Akkermansia muciniphila accelerates intestinal stem cell-mediated epithelial development. Gut Microbes. (2021) 13:1–20. doi: 10.1080/19490976.2021.1892441 PMC7946046 33678130

[31] Peterson LW Artis D. Intestinal epithelial cells: regulators of barrier function and immune homeostasis. Nat Rev Immunol. (2014) 14:141–53. doi: 10.1038/nri3608 24566914

[32] Takiishi T Fenero CIM Câmara NOS. Intestinal barrier and gut microbiota: Shaping our immune responses throughout life. Tissue Barriers. (2017) 5:e1373208. doi: 10.1080/21688370.2017.1373208 28956703 PMC5788425

[33] Zhang Z Shi X Ji J Guo Y Peng Q Hao L. Dihydroartemisinin increased the abundance of Akkermansia muciniphila by YAP1 depression that sensitizes hepatocellular carcinoma to anti-PD-1 immunotherapy. Front Med. (2023) 17:729–46. doi: 10.1007/s11684-022-0978-2 37121958

[34] Wang D-R Wu X-L Sun Y-L. Therapeutic targets and biomarkers of tumor immunotherapy: response versus non-response. Signal Transduct Target Ther. (2022) 7:331. doi: 10.1038/s41392-022-01136-2 36123348 PMC9485144

[35] Bilotta AJ Ma C Yang W Yu Y Yu Y Zhao X. Propionate enhances cell speed and persistence to promote intestinal epithelial turnover and repair. Cell Mol Gastroenterol Hepatol. (2021) 11:1023–44. doi: 10.1016/j.jcmgh.2020.11.011 PMC7898181 33238220

[36] Liu H Wang J He T Becker S Zhang G Li D. Butyrate: A double-edged sword for health? Adv Nutr. (2018) 9:21–9. doi: 10.1093/advances/nmx009 PMC6333934 29438462

[37] Woods DM Sodré AL Villagra A Sarnaik A Sotomayor EM Weber J. HDAC inhibition upregulates PD-1 ligands in melanoma and augments immunotherapy with PD-1 blockade. Cancer Immunol Res. (2015) 3:1375–85. doi: 10.1158/2326-6066.CIR-15-0077-T PMC4674300 26297712

[38] Zhu X Li K Liu G Wu R Zhang Y Wang S. Microbial metabolite butyrate promotes anti-PD-1 antitumor efficacy by modulating T cell receptor signaling of cytotoxic CD8 T cell. Gut Microbes. (2023) 15:2249143. doi: 10.1080/19490976.2023.2249143 37635362 PMC10464552

[39] Li X Su X Liu R Pan Y Fang J Cao L. HDAC inhibition potentiates anti-tumor activity of macrophages and enhances anti-PD-L1-mediated tumor suppression. Oncogene. (2021) 40:1836–50. doi: 10.1038/s41388-020-01636-x PMC7946638 33564072

[40] Coutzac C Jouniaux J-M Paci A Schmidt J Mallardo D Seck A. Systemic short chain fatty acids limit antitumor effect of CTLA-4 blockade in hosts with cancer. Nat Commun. (2020) 11:2168. doi: 10.1038/s41467-020-16079-x 32358520 PMC7195489

[41] Smith PM Howitt MR Panikov N Michaud M Gallini CA Bohlooly-Y M. The microbial metabolites, short-chain fatty acids, regulate colonic Treg cell homeostasis. Science. (2013) 341:569–73. doi: 10.1126/science.1241165 PMC3807819 23828891

[42] Furusawa Y Obata Y Fukuda S Endo TA Nakato G Takahashi D. Commensal microbe-derived butyrate induces the differentiation of colonic regulatory T cells. Nature. (2013) 504:446–50. doi: 10.1038/nature12721 24226770

[43] Arpaia N Campbell C Fan X Dikiy S van der Veeken J deRoos P. Metabolites produced by commensal bacteria promote peripheral regulatory T-cell generation. Nature. (2013) 504:451–5. doi: 10.1038/nature12726 PMC3869884 24226773

