# Supplementary material for: Revealing gut microbiota biomarkers associated with melanoma immunotherapy response and key bacteria-fungi interaction relationships: evidence from metagenomics, machine learning, and SHAP methodology
Source: Front Immunol. 2025 Mar 18;16:1539653. doi: 10.3389/fimmu.2025.1539653 (PMC11959079; doi:10.3389/fimmu.2025.1539653)
Supplement: Supplementary file 1 [file Table1.docx]

Supplementary Material

# Supplementary Tables

**Supplementary Table 1.** The LEfSe identified the most significantly different bacterial taxa between responders and non-responders.

| **Taxa** | **Group** | **LDA** | **pval** |
| --- | --- | --- | --- |
| g__*Segniliparus* | R | 2.424330056 | 0.0263730634558 |
| g__*Megamonas* | R | 2.716138919 | 0.00757920403926 |
| g__*Xanthovirga* | NR | 2.286221944 | 0.0419349268974 |
| g__*Romboutsia* | R | 2.859260855 | 0.000433888945117 |
| g__*Jonquetella* | NR | 2.498402136 | 0.0140788164919 |
| g__*Fannyhessea* | R | 2.050225177 | 0.0423839386326 |
| g__*Mesoaciditoga* | R | 2.325590012 | 0.0344908905371 |
| g__*Akkermansia* | R | 3.998919774 | 0.0404981116446 |
| g__*Holdemanella* | R | 3.109956008 | 0.00276161293545 |
| g__*Aggregatilinea* | R | 2.450584228 | 0.00231291369414 |
| g__*Centipeda* | R | 2.048355587 | 0.000583426587189 |
| g__*Laceyella* | R | 2.210314052 | 0.0212609685141 |
| g__*Phascolarctobacterium* | R | 2.920540681 | 0.017069595 |
| g__*Pigmentiphaga* | NR | 2.213713995 | 0.0485626405101 |
| g__*Thermosynechococcus* | R | 2.037130844 | 0.0137435806436 |
| g__*Rothia* | R | 2.073661206 | 0.0222772573099 |
| g__*Candidatus Desulforudis* | R | 2.224213535 | 0.0466948774552 |
| g__*Parvimonas* | NR | 2.244466968 | 0.0314237770904 |
| g__*Collinsella* | R | 3.881190586 | 0.0378840206472 |
| g__*Colwellia* | R | 2.1094629 | 0.00608284463653 |
| g__*Heliobacillus* | R | 2.31526039 | 0.0125283902356 |
| g__*Mucispirillum* | R | 2.254221483 | 0.0216452828987 |
| g__*Lacticaseibacillus* | R | 2.272632371 | 0.0429093720707 |
| g__*Chlamydia* | R | 2.215558146 | 0.0205300661511 |
| g__*Christensenella* | R | 2.190599547 | 0.0293212691215 |
| g__*Niastella* | R | 2.061202605 | 0.0297006527323 |
| g__*Pontibacillus* | R | 2.012115765 | 0.0491783747802 |
| g__*Endomicrobium* | R | 2.145197338 | 0.00609634693759 |
| g__*Demequina* | R | 2.202733895 | 0.003137023 |
| g__*Viridibacillus* | R | 2.198696225 | 0.0429998072439 |
| g__*Anaerostipes* | R | 2.860095955 | 0.0189035960186 |
| g__*Fusicatenibacter* | R | 2.288970314 | 0.0375675797967 |
| g__*Desulfovirgula* | R | 2.369767917 | 0.028423742 |
| g__*Fenollaria* | R | 2.283623233 | 0.0428758832921 |
| g__*Anaerobutyricum* | R | 3.10250343 | 0.0252520368333 |
| g__*Roseiflexus* | NR | 2.277260388 | 0.0125617062364 |
| g__*Microbacter* | R | 2.059592366 | 0.0207713578999 |
| g__*Candidatus Moduliflexus* | R | 2.016192646 | 0.0054041483393 |
| g__*Turicibacter* | R | 2.451952782 | 0.0108770861486 |
| g__*Streptomyces* | R | 2.256169434 | 0.0369414416704 |
| g__*Candidatus Frackibacter* | R | 2.170336885 | 0.0477617079518 |
| g__*Desulfomonile* | R | 2.229026239 | 0.0346104541016 |
| g__*Faecalibacillus* | R | 2.098527479 | 0.0375675797967 |
| g__*Candidatus Competibacter* | R | 2.244923874 | 0.00457147828464 |
| g__*Dorea* | R | 3.406391766 | 0.023724224 |

LEfSe, Linear discriminant analysis Effect Size; LDA, linear discriminant analysis; R, responder; NR, non-responder. “g_” is genus.

**Supplementary Table 2.** Evaluation of bacterial RF model performance in the test set.

| **Metric** | **Test Value** |
| --- | --- |
| Accuracy | 0.577777778 |
| F1 Score | 0.583662408 |
| Sensitivity | 0.535714286 |
| Specificity | 0.647058824 |
| ROC AUC | 0.636554622 |

RF, random forest; ROC, receiver operating characteristic; AUC, area under the curve.

**Supplementary Table 3.** The LEfSe identified the most significantly different fungal taxa between responders and non-responders.

| **Taxa** | **Group** | **LDA** | **pval** |
| --- | --- | --- | --- |
| g__*Rasamsonia* | R | 2.967104352 | 0.0250125943254 |
| g__*Zancudomyces* | NR | 3.332075772 | 0.0399444965307 |
| g__*Rutstroemia* | R | 3.631575708 | 0.0157133574556 |
| g__*Ganoderma* | R | 2.950972115 | 0.0316681550054 |

LEfSe, Linear discriminant analysis Effect Size; LDA, linear discriminant analysis; R, responder; NR, non-responder. “g_” is genus.

**Supplementary Table 4.** Evaluation of fungal RF model performance in the test set.

| **Metric** | **Test Value** |
| --- | --- |
| Accuracy | 0.488888889 |
| F1 Score | 0.488888889 |
| Sensitivity | 0.392857143 |
| Specificity | 0.647058824 |
| ROC AUC | 0.654411765 |

RF, random forest; ROC, receiver operating characteristic; AUC, area under the curve.

**Supplementary Table 5.** Evaluation of merged RF model performance in the test set.

| **Metric** | **Test Value** |
| --- | --- |
| Accuracy | 0.644 |
| F1 Score | 0.648 |
| Sensitivity | 0.679 |
| Specificity | 0.588 |
| ROC AUC | 0.707 |

RF, random forest; ROC, receiver operating characteristic; AUC, area under the curve.
